# Melone’s concept revisited in comminuted distal radius fractures: the three-dimensional CT mapping

**DOI:** 10.1186/s13018-020-01739-x

**Published:** 2020-06-16

**Authors:** Shuang Li, Ying-Qi Zhang, Gu-Heng Wang, Kai Li, Jian Wang, Ming Ni

**Affiliations:** 1 Department of Orthopaedic Surgery, Pudong New Area Peoples’ Hospital affiliated to Shanghai University of Medicine and Health Sciences, No. 490 Chuanhuan South Road, Pudong New Area, Shanghai, 201299 People’s Republic of China; 2 Department of Orthopaedic Surgery, Tongji Hospital, Tongji University, 389 Xincun Road, Putuo District, Shanghai, 200065 People’s Republic of China; 3 Department of Hand Surgery, Affiliated Hospital of Nantong University, 20 Xisi Road, Nantong City, 226001 Jiangsu Province, People’s Republic of China

**Keywords:** Melone’s concept, Dorsal medial fragment, Volar medial fragment, Fracture mapping, Distal radius fracture

## Abstract

**Background:**

There is no consensus in the literature about the ideal classification of the distal radius fracture for the clinical practice. The traditional Melone classification system divides the distal radius into four basic components, the shaft, radial styloid, dorsal medial fragment, and volar medial fragment. The aim of this study was to identify fracture lines in comminuted distal radius fractures using three-dimensional mapping of computed tomography (CT) images to test the hypothesis that fracture fragments can be divided according to the Melone classification.

**Methods:**

Fifty-nine consecutive OTA/AO 23C3 fractures presented at the hospital between January 2018 and October 2019 were retrospectively reviewed. The fracture lines were characterized in the axial, sagittal, and coronal CT planes. After reducing the fractures in a three-dimensional (3D) model, the fracture lines were plotted from the CT images and were then superimposed on one another and oriented to fit a standard template. The area of articular surfaces was measured and compared to quantify the differences between the radial bone fragments.

**Results:**

Thirty-five cases (59.3%) in this study fit the Melone classification and 24 cases (40.7%) did not. On the radiocarpal surface, there was a greater concentration of fracture lines in the dorsal area of the radius than in the volar area. On the distal radioulnar joint (DRUJ), the fracture lines were focused around two specific concentrated regions. For the articular surface area, the mean area of the radial styloid, volar medial fragment, and dorsal medial fragment was 141.13 ± 90.16 mm^2^, 147.79 ± 75.94 mm^2^, and 79.05 ± 70.73 mm^2^, respectively. There was a significant difference in articular surface area for the Melone fragments (*P* = 0.002).

**Conclusions:**

The Melone classification system is not suitable for characterizing all C3 fractures. The findings of this study confirm that the dorsal medial fragments are relatively comminuted and small. Extra care should be given to these small fragments when reducing the fracture.

## Background

Distal radius fractures (DRFs) are the most common bone fractures in adults [1, 2]. Approximately half of all DRFs occur across the articular surface, and the majority of these are complete articular fractures (Orthopaedic Trauma Association/Arbeitsgemeinschaftfür Osteosynthesefragen classification type C, AO/OTA type C) [3]. Of these intra-articular fractures, a C3 fracture is the most complex, indicating extensive injury to the surfaces of the radiocarpal joint and distal radioulnar joint (DRUJ) [4]. This complexity is due to the comminution at the articular surfaces and metaphys and due to the vulnerable soft tissue envelope around the wrist. In 1984, Melone et al. developed a classification system for fractures of the distal radius, noting that such fractures are frequently bi-articular injuries and are comprised four basic components: radial shaft, radial styloid, the dorsal medial fragment, and volar medial fragment [5]. However, the Melone classification is based on X-ray data rather than accurate physiological measurements [3]. The other classifications such as Frykman classification and Fernandez classification do not suffice for individual communication-related use in daily practice [6].

Fracture mapping is an imaging method used to examine fracture characteristics and morphology [7]. It allows for the quantitative evaluation of fractures based on CT images and has already been used for investigating scapular fractures, Hoffa fractures and distal radius fractures [7–10]. The aim of this study is to use fracture mapping on distal radius fractures of AO/OTA type 23C3 and test the null hypothesis that fracture fragments can be divided according to Melone’s classification (shaft, radial styloid, the dorsal medial fragment, and volar medial fragment) [5].Given the limited information available on the anatomical patterns of articular comminution in C3 fractures, this study also aimed to introduce heat mapping of fracture lines to evaluate the morphology of comminuted distal radius fractures.

## Materials and methods

Sixty-nine consecutive patients with intra-articular distal radius fractures were enrolled at Pudong New Area Peoples’ Hospital affiliated to Shanghai University of Medicine and Health Sciences between January 2018 and October 2019. The study was approved by the hospital’s human subject research ethics committee, and data collection and analysis were performed in compliance with the Declaration of Helsinki. All subjects were verbally briefed on the purpose and methods in this study and gave informed consent prior to participation. All patients underwent a CT examination, with the result that 2 patients were excluded due to insufficient quality of images and 8 patients were excluded because they did not have a C3 type fracture. Inclusion criteria were a closed AO/OTA type 23C3 distal radius fracture, age ≥ 18 years, and availability of radiographic and CT images. Patients were excluded if they had an open fracture, a history of surgery to the wrist or hand, or any contraindication to casting such as edema or poor skin condition.

Fifty-nine patients with AO/OTA type 23C3 fractures were included in the final study. The patient group comprised 26 men and 33 women ranging in age from 18 to 69 years (average age 50.1 years). There were 28 fractures on the left hand and 31 fractures on the right hand. A 64-slice CT (Philips, Brilliance 64) was taken of the fracture region for each patient with the forearm in a neutral position. Images were obtained in the axial plane with a slice thickness of 0.625 mm, and all data was saved as digital imaging and communications in medicine (DICOM) data. The DICOM data was then reconstructed into 3D images using the CT bone segmentation function in Mimics soft (version 18.0, Materialise NV Technologielaan, Leuven, Belgium). A threshold of 250 Hounsfield units was used to identify fracture fragments.

**Fig. 1:**
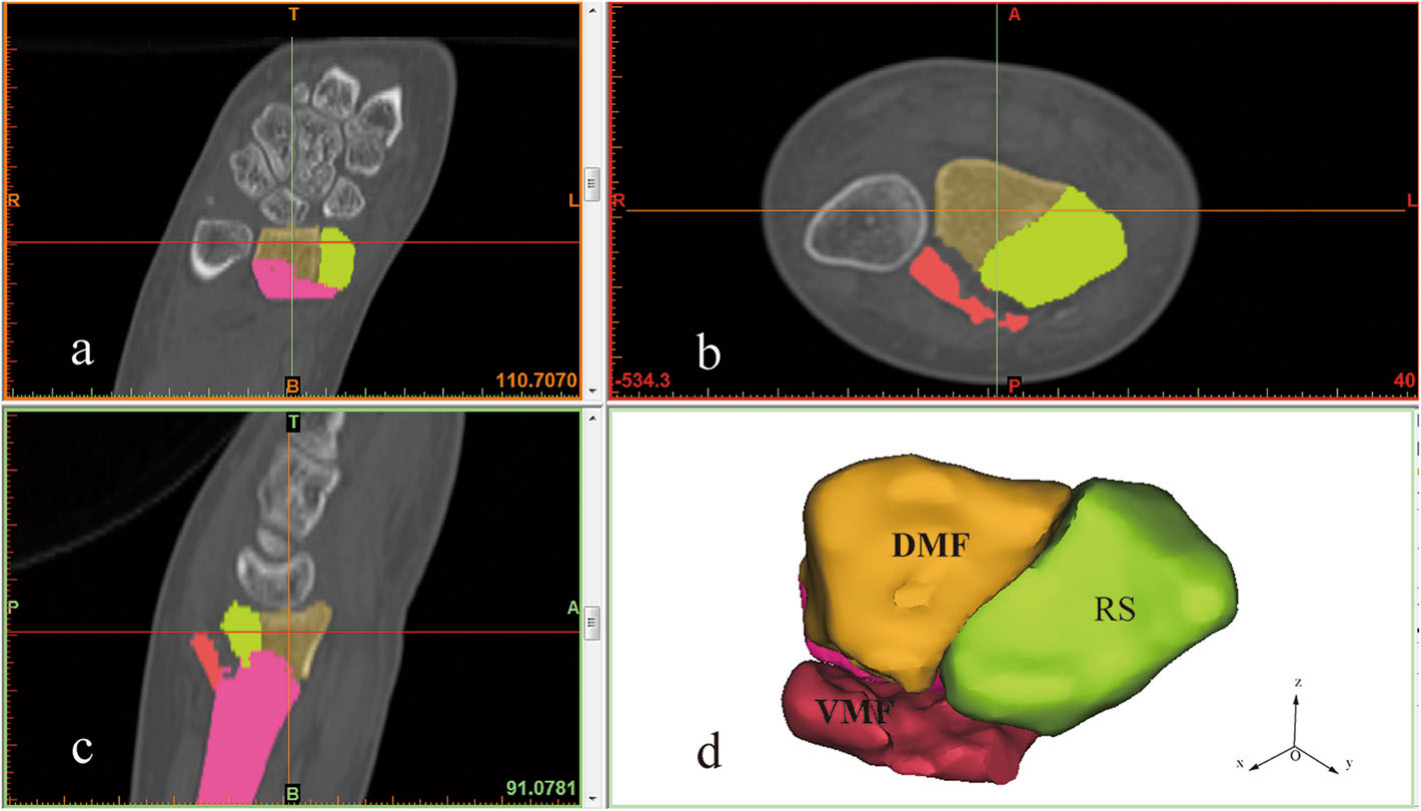
3D reconstructionof distal radius fracture: **a** coronal plane; **b** sagittal plane; **c** axial plane; **d** 3D model. RS-radial styloid, DMF- dorsal medial fragment, VMF-volar medial fragment

As shown in Fig. 1, the fracture fragments were characterized in the axial, sagittal, and coronal CT planes. The coordinate system of the model was standardized to the *x*-, *y*-, and *z*-axes in Mimics. The Lister tuber was the origin point, with the *x*-axis oriented in the radial–ulnar direction, the *y*-axis in the volar–dorsal direction, and the z-axis in the proximal–distal direction. Using this 3D model, the displacement between bone fragments could be measured. After recording the fracture gaps, the fragments were reduced and the 3D models were exported to the 3-Matic software (version 10.0, Materialise NV Technologielaan, Leuven, Belgium) for further analysis. All 31 models of right-sided radii were mirrored to make the orientation match that of the left-sided radii.

**Fig. 2:**
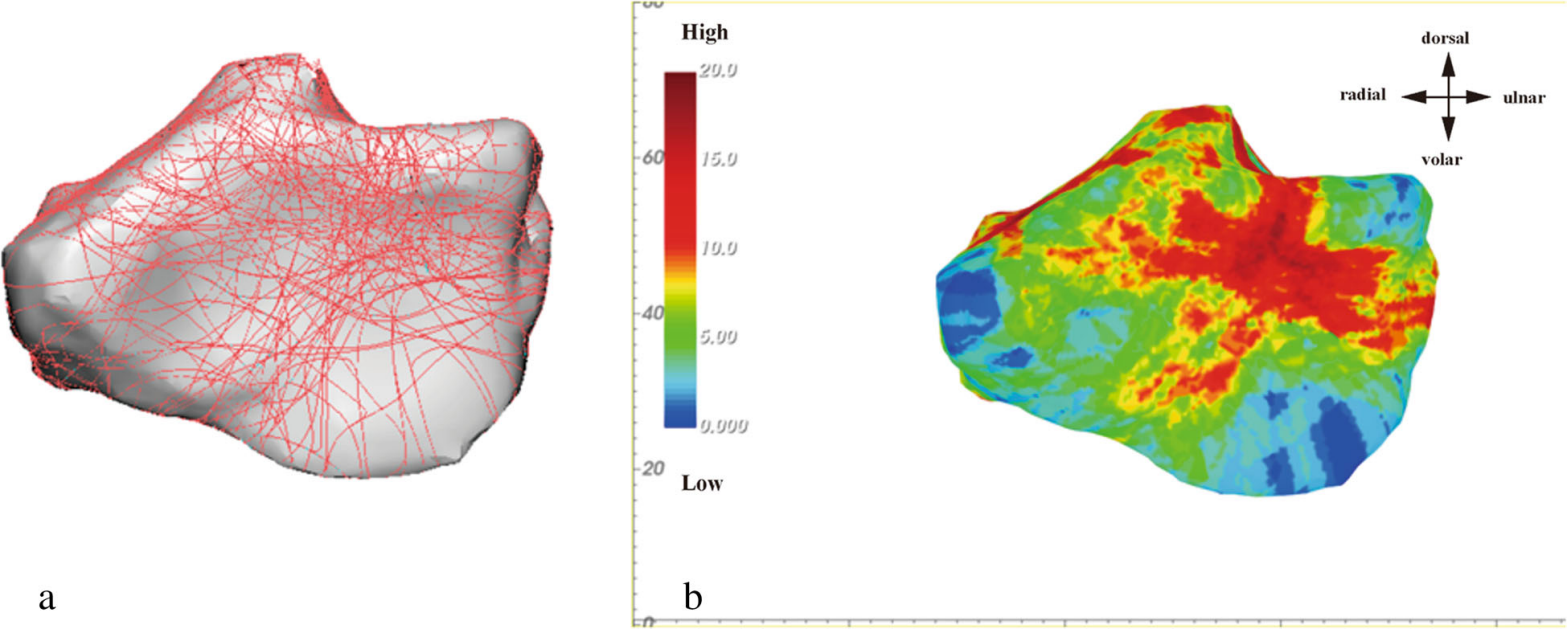
The distribution and heat mapping of the fracture lines in the comminution zone of the intra-articular distal radius fractures (DRFs): **a** Fracture lines were distributed on the distal radius surface. **b** Fracture line density was color-coded from blue (low density, zero fracture lines) to deep red (high density, 20 fracture lines crossing the region)

Individual maps of all 59 DRFs were first created, which were then used to create a single color-coded fracture map using the process described below. Fracture line density was color-coded from blue (low density) to deep red (high density) (Fig. 2b). On the radiocarpal joint surface, the heat map of the C3 fracture shows a relatively intact volar medial fragment (green) and a high concentration of fractures on the dorsal ridge of the lunate fossa (red).

**Fig. 3:**
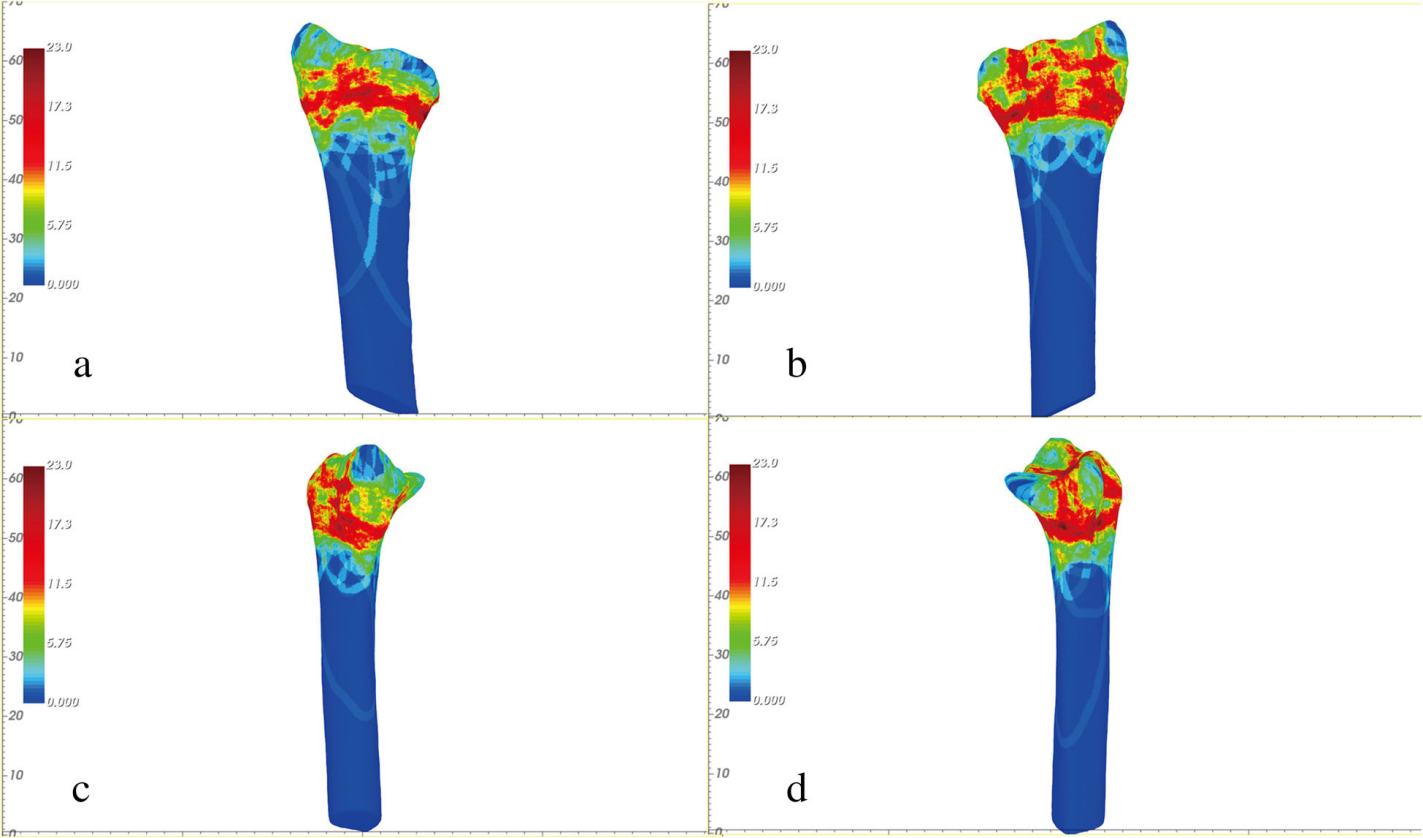
The heat map of the distal radii: **a** On the volar view, the fracture lines were mainly located on the volar cortex and had an arc-like appearance. **b** On the dorsal view, the fracture lines were concentrated around the circumference of the Lister tubercle. **c** View of the radial styloid process showing the fracture lines around radial styloid process. **d** View of the distal radio-ulnar joint (DRUJ) showing concentrations of fracture lines around two specific regions of the middle and dorsal side in the sigmoid notch

The orientation and alignment of anatomical landmarks (radial styloid, sigmoid notch, and volar cortex) were standardized across all models to allow for direct comparison. After plotting the fracture lines for each individual DRF, these individual maps were superimposed to create a compilation heat map showing the density of fracture lines (width = 3 mm). Figure 3 shows the heat map of the distal radii.

Patient characteristics and fracture measurements are summarized as means and standard deviations. Comparisons between fragments were performed using GraphPad Prism 6.0 with a one-way analysis of variance (one-way ANOVA). A *P* value of < 0.05 was considered statistically significant.

## Results

### Patient demographics and fracture characteristics

As detailed in Table 1, among the subgroups of C3 fractures, the majority were type C3.2 (*n* = 40). The fractures were characterized by radial inclination, volar tilt and radial length. In the subgroups, there was no significant difference in radial inclination (*P* = 0.15) and volar tilt (*P* = 0.82). There was a significant difference in radial length (*P* = 0.005).

**Table 1:** Patient demographics and fracture characteristics

Fracture type	23-C3.1	23-C3.2	23-C3.3	*P value*
Quantity	7	40	12	–
Age	20 (18–22)	51.2 (21–69)	52.7 (40–64)	–
Sex				
Male	3	16	6	–
Female	4	24	6	–
Side of injury				
Left hand	2	15	5	–
Right hand	5	25	7	–
Radial inclination (°)	21.1 ± 0.5	15.2 ± 5.6	18.9 ± 7.7	0.15
Volar tilt (°)	− 10.1 ± 17.3	− 7.4 ± 14.6	− 3.5 ± 19.8	0.82
Radial length (mm)	13.6 ± 2.5	7.6 ± 2.5	6.6 ± 2.9	0.005

### Fracture fragment articular surface area

If the displacement between two fracture fragments was less than 1 mm, then this was not regarded as a fracture in this study and the particular bone section was considered intact. As shown in Table 2, of the 59 C3 fractures in this study, 35 cases (59.3%) fit within Melone’s classification and 24 cases (40.7%) did not. A total of 59 C3 fractures created 237 articular fragments, with 139 being classified as Melone fragments and 98 falling outside this classification. Among the Melone fractures, the volar medial fragments had the highest ratio of intact fragments (88.6%), followed by the radial styloid (80%), and dorsal medial (42.9%). Therefore, the volar medial fragment was the most intact, and the dorsal medial fragment was the most comminuted.

**Table 2:** Baseline fracture fragment characteristics after reduction in 3D model

	Intact	Double split	Triple split	Comminution
C3 Melone fragments (*n* = 35)				
Radial styloid	28	4	1	2
Volar medial	31	2	1	1
Dorsal medial	15	12	3	5
C3 fractures that did not fit Melone (*n* = 24)				
Radial styloid	2	13	0	6
Volar medial	11	4	0	5
Dorsal medial	4	8	3	7
Radial styloid connected to volar medial fragment^a^	13	–	–	–
Radial styloid connected to dorsal medial fragment^b^	2	–	–	–
High coronal/die punch	5	–	–	–
Central fragment	5	–	–	–

^a^Model including repetition count

^b^Model including repetition count

It can be seen that the area of the radial styloid fragment was 170.7 ± 111.7 mm^2^ [range 17.49–451.3 mm^2^], the area of the volar medial fragment was 165.3 ± 100.4 mm^2^ [range 11.45–451.3 mm^2^], and the area of the dorsal medial fragment was 86.85 ± 78.16 mm^2^ [range 10.21–367.3 mm^2^]. There was a significance difference in articular surface area among the Melone fragments (*P* = 0.002). The articular surface area of the radial styloid occupied about 40.2% of the total radial-carpal surface area, with the volar medial side accounting for 39.1% and dorsal medial accounting for 20.6%. This equates to a ratio of roughly 2:2:1, which means that the dorsal articular surface covers 20% of total surface area and is the smallest region of the radius.

## Discussion

Severely comminuted intra-articular fractures of the distal radius can be challenging to treat. Restoration of congruity of the articular surface, radial length, radial tilt angle, and volar tilt is important for a good clinical result. Failure to achieve and maintain anatomic restoration can lead to post-traumatic arthritis, distal radioulnar joint instability, and ulnar impaction syndrome, with resultant pain, joint stiffness, and decreased motion and strength [11, 12]. These are primarily procedure-related when the surgeon holds a vague recognition of the characteristics of comminuted intra-articular fractures, and the morphology of the fracture fragments [13].

Fractures of the distal radius have many types and classifications. These classifications include the following: Frykman, Melone, AO/OTA, and Fernandez classification [14, 15]. Each type will require a specific understanding of the classification and management of the fractures (not all of them can be treated with a cast). Some classifications do not suffice for individual communication-related use in daily practice (Table 3) [16, 17]. There is no consensus in the literature about the ideal classification of the distal radius fracture for the clinical practice. The Melone system is simply and widely used for classifying the morphology of DRFs. However, the Melone classification system is based on X-ray data rather than accurate physiological measurements. More recently, 3D reconstruction of CT images has been used to assess the comminution of intra-articular DRFs [18]. This study was designed to assess whether the Melone system was suitable for classifying all DRF fragments, especially for comminuted DRFs.

**Table 3:** Pros and cons of the classifications of the distal radius fracture

Classifications	Advantages	Disadvantages
Frykman (1967)	Identifying individual involvement of the radiocarpal and radioulnar as well as the presence or absence of a fracture of the ulnar styloid process	Not making a distinction between displaced and non-displaced intra-articular fractures
Melone (1984)	Creating characteristic fracture fragments based on the effect of the lunate’s impaction on the radial articular surface	Not including the fractures neither affect the articular surface of the radiocarpal nor the radioulnar joints
AO/OTA (1986/2018)	The most detailed classification system to date	Depending on CT and poor reproducibility
Mayo Clinic classification (1992)	Emphasizing the role of specific articular contact areas and the fracture stability	Not quantitative
Fernandez (1993)	Providing the mechanism of injury and potential soft tissue damage (tendon, ligament, nerve, vessels)	Not including the radial articular surface

The results of this study showed that the crossing concentration of fracture lines on the radiocarpal surface and the fracture line concentrated regions of the middle 1/3 and the dorsal 1/3 of the sigmoid notch. On the radiocarpal joint surface, the heat map of the C3 fracture shows a relatively intact volar medial fragment and a high concentration of fractures on the dorsal ridge of the lunate fossa. Also, we found that the dorsal medial fragments were relatively small. The Melone classification does not include the distal radioulnar joint integrity, soft tissue injuries, and Melone component size. To the author’s knowledge, the Melone components were given the most detailed account of articular surface by using fracture mapping to date.

A key finding of this study was that the Melone classification system was not suitable for characterizing all C3 fractures. Teunis et al. reported that the Melone system could allow for up to 76% (38/50) of fractures to be classified, but this current study found that only 59.3% (35/59) of C3 fractures fit within the Melone system [3]. For the Melone groups, the ratio of articular surface of the radial styloid to volar medial to dorsal medial articular area was roughly 2:2:1, meaning the dorsal medial fragments were relatively small. For the non-Melone fractures, Table 2 shows the various other fracture patterns that appeared in this study.

Another key finding of this study is that the fracture lines are mainly located on the dorsal portion of the radius and around Lister’s tuber (Fig. 3). The general approach is to first reduce the fragments and press on Lister’s tuber or use an alternative means to maintain the tuber anatomy, and then secure with a plate or Kirschner wire [19]. It has been reported that alignment of the volar fragment with the radial styloid is more critical than the dorsal fragment as the dorsal fragment may not routinely benefit from specific reduction and fixation [3]. The dorsal fragment is not an isolated articular surface of the distal radius but an important area with dense trabecular bone in the dorsal aspect of the distal radius. Severe intra-articular fractures of the distal radius with comminuted, displaced, and malrotated fragments are difficult to stabilize using standard plates or screws. There are also challenges with treating the fractured joint in cases of substantial cartilage loss and extended metaphyseal/subchondral bony defects [19]. The importance of the dorsal medial fragment is that it helps to restore volar cortical alignment and thus increases the stability after manipulation of distal radial fractures [20]. Reducing and securing the small and comminuted dorsal medial fragments, especially around the articular area, are beneficial for restoring good functionality to the wrist.

The practicality and accuracy of fracture mapping have been reported in a number of studies investigating scapula bone fractures, tibial plateau fractures, radial head fractures, and ulnar pulley articular fractures [21–24]. The radial fracture map developed in this current study can also be used to assess existing fracture classification systems. An accurate map of fracture fragments allows for optimal preoperative planning, which includes the selection of a suitable surgical approach and fixation constructs. The results presented in this study provide insight into fracture morphology, which can assist with fracture classification and the design of biomechanical studies, ultimately aiding treatment.

There are some limitations to this study that should be noted. The first is that the analysis was carried out retrospectively on patients recruited over a short period of time. The second is the patient study group was relatively small, which could have influenced the clinical outcomes. For example, the patients with C3.2 and C3.3 fractures were roughly 3 times older than the patients with C3.1 fractures. The reason is that the sample of C3.1 fractures was so small that the comparability of populations is weakened. Finally, the data was primarily sourced from CT images, so for more accurate results, this should be accessed through by a randomized clinical trial.

In conclusion, we believe that severely comminuted intra-articular (AO/OTA 23C3) distal radial fractures should be assessed using fracture mapping before beginning any invasive treatments. The traditional Melone’s concept is not suitable for characterizing all C3 fractures. The fracture comminution regions of the dorsal fragments should be fixed using a suitable angle and positioning of the screws or Kirschner wire so as to achieve a stable reduction.

## Data Availability

All the data will be available upon motivated request to the corresponding author of the present paper.

## Abbreviations

CT: Computed tomography
OTA/AO: Orthopaedic Trauma Association/Arbeitsgemeinschaftfür Osteosynthesefragen
3D: Three dimensional
DRUJ: Distal radioulnar joint
DRF: Distal radius fracture
DICOM: Digital Imaging and Communications in Medicine
one-way ANOVA: one-way analysis of variance
